# Tumid Lupus Erythematosus Presenting as Patchy Alopecia: A Discussion of Alopecia Associated With Lupus Erythematosus

**DOI:** 10.7759/cureus.36956

**Published:** 2023-03-31

**Authors:** Mason R Henehan, Sydney Stillman, Hailey E Grubbs, Carlos Cohen

**Affiliations:** 1 Dermatology, Broward Health Medical Center, Fort Lauderdale, USA; 2 Internal Medicine, Yale University/Waterbury Hospital, Waterbury, USA

**Keywords:** scalp lesions, hair loss treatment, derm-rheum, non-scarring alopecia, tumid lupus

## Abstract

Tumid lupus erythematosus (TLE), a subtype of chronic cutaneous lupus erythematosus (CCLE), presents with firm erythematous plaques that lack surface changes such as follicular plugging or scale. These lesions most commonly occur on the face and other photosensitive areas but may also present on the scalp as recurrent circumscribed patches of non-cicatricial alopecia. Including TLE as part of the differential for non-cicatricial alopecia can prove helpful in patients who fail to improve with empiric first-line treatments for more common causes of hair loss. We report a case of TLE that clinically mimicked alopecia areata and seek to highlight the relevant clinical and histological features to promote earlier diagnosis of this entity. A discussion of improved diagnostic and treatment modalities, as well as identifying the uncommon but possible association of TLE with underlying systemic disease, adds to the importance of maintaining clinical suspicion for TLE. Finally, we provide an overview to discriminate TLE from other forms of cutaneous lupus and their unique patterns of alopecia when presenting on the scalp.

## Introduction

Tumid lupus erythematosus (TLE), a subtype of chronic cutaneous lupus erythematosus, presents with firm erythematous and edematous plaques that lack surface changes such as follicular plugging or scale [1]. These lesions most commonly occur on the face and other photosensitive areas, including the upper chest, upper back, extensor arms, and shoulders. However, TLE may also present on the scalp as recurrent circumscribed patches of non-cicatricial alopecia and may be more resistant to empiric first-line treatments for more common causes of hair loss, such as alopecia areata. Improved diagnosis and treatment of this chronic disease, as well as identifying the uncommon but possible association of TLE with underlying systemic disease, adds to the importance of maintaining clinical suspicion for early diagnosis and screening [2,3].

## Case presentation

A 39-year-old male with a past medical history of only childhood asthma presented to our dermatology clinic with complaints of relapsing areas of alopecia on the scalp for several months. He was previously diagnosed clinically with alopecia areata and treated with intralesional triamcinolone by another dermatologist.

**Figure 1:**
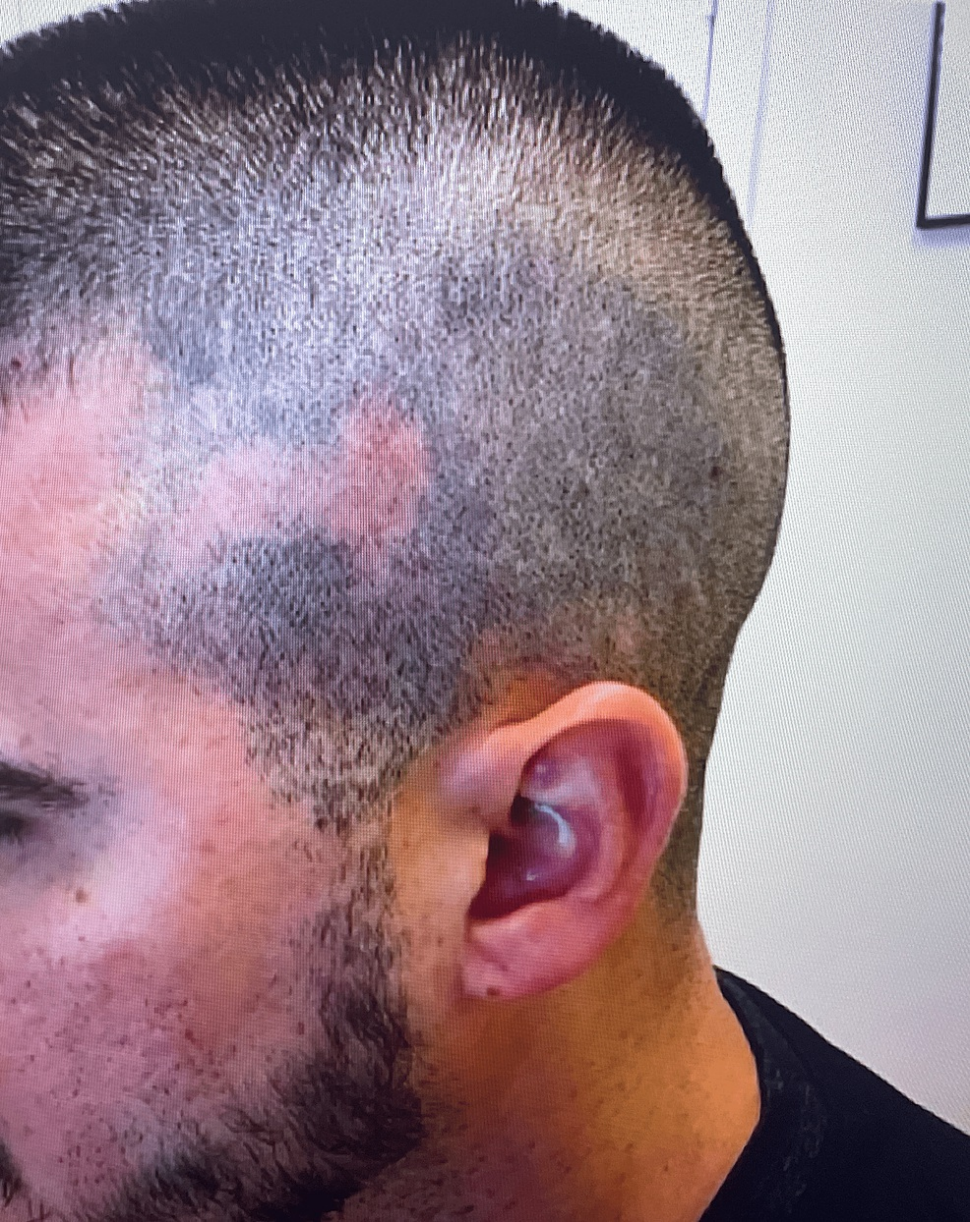
Patient at the time of presentation with a few circumscribed patches of alopecia, some with new hair growth. Mild dermal atrophy was noted overlying the left temporal scalp.

**Figure 2:**
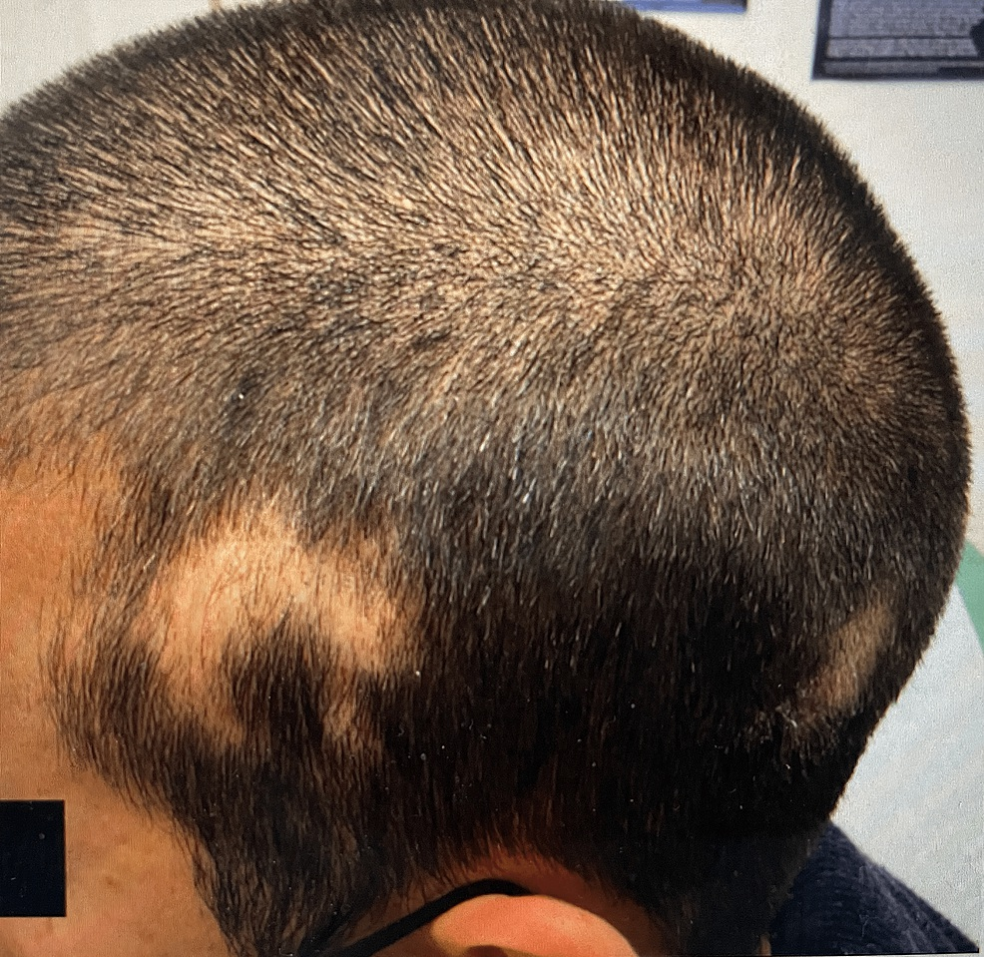
Patient presents eight months later with complaints of recurrent patchy alopecia. A firm, tender nodule with surrounding bogginess was appreciated on palpation.

Physical examination revealed multiple, well-circumscribed areas of patchy alopecia. New hair growth was appreciated in several patches (Figure 1). The patient reported some improvement with prior intralesional triamcinolone, so treatment was continued two more times until satisfactory results were achieved. Eight months later, the patient returned to the office again complaining of alopecia and now reported a slowly enlarging, painful area within one patch of hair loss for the past three weeks (Figure 2). He denied any fevers, weight loss, fatigue, myalgias, arthralgias, or photosensitivity. The patient reported prior sexual activity but did not identify a clear temporal relationship between the onset of the alopecic patches around the time of a new sexual partner. Within a patch of alopecia on the left posterolateral parietal scalp, there was a tender, faintly erythematous plaque with underlying edema and no scale. Trichoscopy revealed yellow dots at the follicular ostia, but no black dots, no tapered or “exclamation point” hairs, no scarring, no follicular dropout, and no perifollicular scale were observed. Additionally, there was associated localized, non-tender lymphadenopathy of the occipital scalp. A 4-mm punch biopsy of the patch of alopecia was later performed; the site had not received intralesional corticosteroid for 14 months at the time of the biopsy. The patient also went for an ultrasound of the lymph node, which revealed no significant pathology.

**Figure 3:**
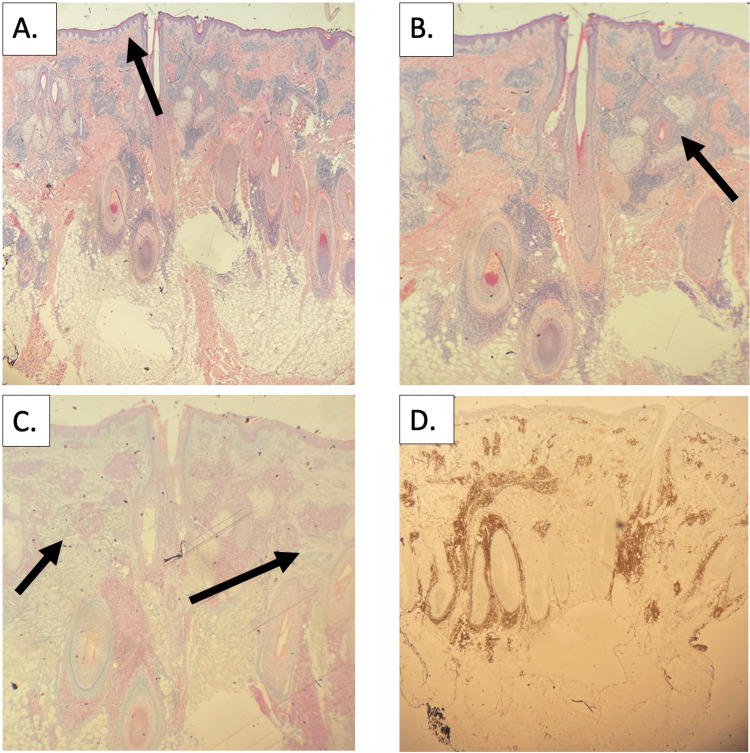
A: Hematoxylin and eosin (2.5× magnification) staining showing lymphocyte-predominant inflammation mainly surrounding periadnexal structures. Some superficial perivascular inflammation is also present but without significant interface changes at the basement membrane (arrow). B: Hematoxylin and eosin (4× magnification) demonstrating periadnexal lymphocytic infiltrate (arrow). C: Alcian blue staining (4× magnification) revealing increased dermal mucin (arrows). D: CD3 staining (2.5× magnification) confirming that the periadnexal inflammation is predominantly composed of T-cells. CD20 staining (not pictured) was also performed and was nearly negative. No significant lymphocytic epidermotropism was identified. CD20: cluster of differentiation 20

The biopsy revealed a superficial and deep dermal perivascular and periadnexal lymphocytic infiltrate (eccrine glands more prominently involved than hair follicles) with increased dermal mucin deposition (Figure 3A-3D). Lymphocyte staining for cluster of differentiation (CD) 3 (CD3) was positive, while CD20 was negative. Given the dermal mucinosis and observation that lymphocytic infiltrates were not limited to the peribulbar region, further staining to distinguish from alopecia areata was not pursued in this case. The absence of lymphocyte epidermotropism in the hair follicle epithelium suggested against folliculotropic mycosis fungoides. Serology was negative for antinuclear antibody (ANA), anti-double-stranded deoxyribonucleic acid (anti-dsDNA), anti-Smith/ribonucleoprotein (RNP), and anti-Sjögren’s syndrome-related antigen A (anti-SSA)/Sjögren’s syndrome-related antigen B (SSB). Complement C3 and C4 levels were normal. Rapid plasma reagin was also negative. Based on the clinicopathologic correlation, a diagnosis of tumid lupus erythematosus was made. Direct immunofluorescence (DIF) was not pursued in this case given that TLE typically has negative immunofluorescence, ANA was negative, and no clinical features of another lupus subtype (scale, scarring, dyspigmentation, malar rash, etc.) nor findings of another connective tissue disease were observed.

**Figure 4:**
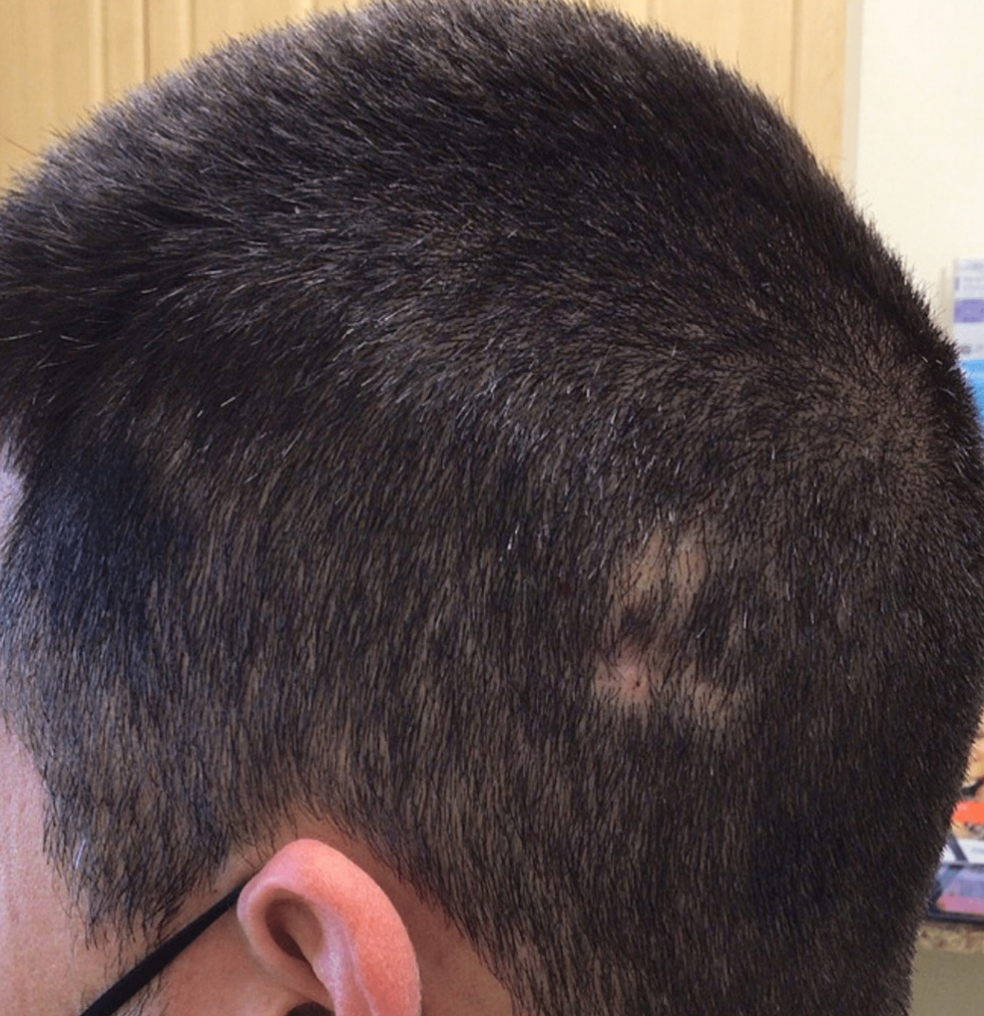
Patient three months after the final diagnosis, now with one residual patch of alopecia. At this time, the patient had been on hydroxychloroquine for two months.

The patient was started on topical tacrolimus and oral hydroxychloroquine 200 mg twice daily, while intralesional triamcinolone injections were continued. General sun protection with clothing and sunscreen was advised, with attention to using a broad-brim hat or other accessories for scalp protection as hair regrowth occurs. The tender nodule and boggy scalp resolved three months later, and gradually, his patches of alopecia demonstrated hair regrowth (Figure 4).

## Discussion

TLE is an entity where the key difference from other subtypes of cutaneous lupus erythematosus is that inflammation is predominantly dermal. Accordingly, skin lesions lack surface epidermal changes such as scale, ulceration, or crust and commonly heal without scarring or any significant dyspigmentation [2]. This description is consistent with our patient, although in the unique location of the scalp as opposed to more classic areas on the upper trunk and arms. Regarding the risk of systemic lupus erythematosus (SLE), there have been reports of systemic involvement with TLE, but this is much less common than other forms of cutaneous lupus [2]. Due to its unique relapsing and remitting course with lower rates of systemic involvement, a shift toward a unique classification for TLE as “intermittent cutaneous lupus erythematosus” has been considered [4]. This intermittent pattern without underlying systemic disease was also true for our patient’s clinical course.

The histology of TLE reflects the pertinent clinical differences described above in that there is minimal epidermal involvement or interface change, along with a lack of follicular plugging or hyperkeratosis. TLE is associated with superficial and deep perivascular lymphocytic infiltrates with prominent eccrine gland involvement and dense mucin deposition. CD3 staining will demonstrate a T-cell-predominant lymphocytic infiltrate [3]. Many findings seen in other subtypes of cutaneous lupus, including vacuolar degeneration and thickening of the basement membrane, are absent in TLE. Direct immunofluorescence is often negative.

The clinical presentation and histological features of TLE are often sufficient for distinguishing it from other forms of cutaneous lupus. However, lesions of TLE localized to the scalp may prove challenging to differentiate from other causes of non-cicatricial alopecia such as alopecia areata, secondary syphilis, trichotillomania, telogen or anagen effluvium, or lupus hair (a sign that can be seen in patients with SLE). Thus, approaching alopecia in an algorithmic manner can be beneficial for accurate assessment. Determining whether or not the alopecia is cicatricial and recognizing patterns of distribution are the first steps. Using this system, TLE will produce non-cicatricial foci of alopecia typically in a circumscribed pattern. Hence, discoid lupus erythematosus (DLE) is often easily distinguished from TLE due to its scarring nature with follicular dropout on trichoscopy, as well as prominent dyspigmentation [5]. In the remainder of our discussion, we will focus on common non-cicatricial forms of alopecia with a focus on distinguishing acute cutaneous lupus erythematosus (ACLE), subacute cutaneous lupus (SCLE), and TLE alongside their unique histopathologic features [6].

Both TLE and alopecia areata may present similarly with non-scarring, round, circumscribed patches of hair loss. The presence of subtle erythema or edema within the foci of alopecia can be more suggestive of TLE, while ophiasis pattern (hair loss along the parieto-temporo-occipital region) can be suggestive of alopecia areata. Trichoscopic changes such as yellow dots, black dots, exclamation point hairs, short vellus hairs, and tapered hairs are among the most commonly reported in alopecia areata but are nonspecific as isolated features [7]. Trichoscopy of TLE is poorly described in the literature due to a paucity of cases focused on the scalp. Since specific clinical findings often are not present, a skin biopsy with histological examination is usually needed to distinguish these entities. In contrast to TLE, the histopathology of alopecia areata will show a mononuclear cell infiltrate localized around anagen hair bulbs. SCLE can also present with patchy hair loss. However, SCLE will typically have more obvious inflammatory skin surface findings with annular papulosquamous or psoriasiform lesions with a significant overlying scale. Biopsy on hematoxylin and eosin (H&E) shows intense interface lymphocytic inflammation, foci of keratinocyte dyskeratosis, and psoriasiform hyperplasia of the epidermis. On direct immunofluorescence, well-established lesions may show foci of a “full house” pattern of granular positivity for multiple immunoglobulin types at the dermal-epidermal junction [6].

Regarding non-cicatricial alopecia in a diffuse pattern, this can be seen in alopecia related to ACLE. Other entities in this differential include alopecia universalis, anagen effluvium (AE), and telogen effluvium (TE). Due to the strong association between ACLE and systemic involvement, a thorough history and physical examination looking for findings such as a malar rash can be helpful. The histology of classic ACLE lesions reveals a mild interface dermatitis with subtle vacuolar degeneration of the dermal-epidermal junction, and biopsy of the scalp may show nonspecific findings. Hence, a biopsy for direct immunofluorescence may be an additional tool for dermatologists in looking for the positive lupus band and/or in vivo ANA. The diagnosis of AE and TE will also be primarily clinical. Although nonspecific, AE hairs will show a tapered fracture of the hair shaft with black dots and exclamation mark hairs on trichoscopy, with normal pigmentation of the hair shaft. TE demonstrates a characteristic short, club-shaped root with depigmentation of the proximal part of the hair shaft. Biopsy of AE reveals a normal anagen-to-telogen ratio of hair follicles, whereas biopsy of TE shows greater than 15% of follicles in the telogen phase. Clinical-pathological correlation is important here, as alopecia areata also has a significant phase shift of hairs but will present differently clinically, in a circumscribed rather than diffuse pattern [8].

Regarding non-cicatricial alopecia in a frontotemporal pattern, a manifestation of systemic lupus erythematosus (SLE) known as “lupus hair” can present in this pattern. These patients most commonly present with hair that is dry and fragile, and with frontal scalp thinning. Lupus hair may occasionally have features of other morphological forms of cutaneous lupus as described above [6]. Other entities in the differential of the frontotemporal pattern would be androgenetic alopecia, traction alopecia, and frontal fibrosing alopecia, as the early stages of these slowly progressive diseases may have minimal erythema or scarring despite the possible progression to permanent hair loss and/or scarring if unaddressed.

## Conclusions

In summary, when a patient presents with non-scarring alopecia, the differential can be extensive. With an algorithmic approach, diagnosis can often be made clinically with a thorough physical examination and trichoscopic evaluation. A biopsy is warranted when lesions have unusual clinical features or relapse frequently in response to standard therapies. As a general rule in medicine, when common diagnoses prove resistant to first-line treatments, the astute clinician should pause to reconsider if the clinical diagnosis is correct and potentially pursue further diagnostic workup. The diagnosis of TLE is critical, as these patients should be screened for systemic involvement and are candidates to try antimalarial medications such as hydroxychloroquine, in addition to standard therapies such as topical tacrolimus or intralesional triamcinolone. In conclusion, keeping an open mind to alternate diagnoses in alopecia may prevent delayed diagnoses, improve treatment success, and minimize both disease progression and patient distress.
